# Nonlinearities distribution Laplace transform-homotopy perturbation method

**DOI:** 10.1186/2193-1801-3-594

**Published:** 2014-10-09

**Authors:** Uriel Filobello-Nino, Hector Vazquez-Leal, Brahim Benhammouda, Luis Hernandez-Martinez, Claudio Hoyos-Reyes, Jose Antonio Agustin Perez-Sesma, Victor Manuel Jimenez-Fernandez, Domitilo Pereyra-Diaz, Antonio Marin-Hernandez, Alejandro Diaz-Sanchez, Jesus Huerta-Chua, Juan Cervantes-Perez

**Affiliations:** Electronic Instrumentation and Atmospheric Sciences School, Universidad Veracruzana, Circuito Gonzalo Aguirre Beltrán S/N, Xalapa, 9100 Veracruz México; Higher Colleges of Technology, Abu Dhabi Men’s College, P.O. Box 25035, Abu Dhabi, United Arab Emirates; National Institute for Astrophysics, Optics and Electronics, Luis Enrique Erro #1, Sta. María Tonantzintla, 72840 Puebla, México; Department of Artificial Intelligence, Universidad Veracruzana, Sebastián Camacho No. 5 Centro., Xalapa, Veracruz 91000 México; Civil Engineering School, Universidad Veracruzana, Venustiano Carranza S/N, Col. Revolución, 93390 Poza Rica, Veracruz México

**Keywords:** Homotopy perturbation method, Nonlinear differential equation, Approximate solutions, Laplace transform, Laplace transform homotopy perturbation method, Finite boundary conditions

## Abstract

This article proposes non-linearities distribution Laplace transform-homotopy perturbation method (NDLT-HPM) to find approximate solutions for linear and nonlinear differential equations with finite boundary conditions. We will see that the method is particularly relevant in case of equations with nonhomogeneous non-polynomial terms. Comparing figures between approximate and exact solutions we show the effectiveness of the proposed method.

## Introduction

Laplace Transform (L.T.) (or operational calculus) has played an important role in mathematics (Murray 1988), not only for its theoretical interest, but also because such method allows to solve, in a simpler fashion, many problems in science and engineering, in comparison with other mathematical techniques (Murray 1988). In particular the L.T. is useful for solving ODES with constant coefficients, and initial conditions, but also can be used to solve some cases of differential equations with variable coefficients and partial differential equations (Murray 1988). On the other hand, applications of L.T. for nonlinear ordinary differential equations mainly focus to find approximate solutions, thus in reference (Aminikhan & Hemmatnezhad 2012) was reported a combination of Homotopy Perturbation Method (HPM) and L.T. method (LT-HPM), in order to obtain highly accurate solutions for these equations. However, just as with L.T; LT-HPM method has been used mainly to find solutions to problems with initial conditions (Aminikhan & Hemmatnezhad 2012; Aminikhah 2012), because it is directly related with them. Therefore (Filobello-Nino et al. 2013) presented successfully, the application of LT-HPM, in the search for approximate solutions for nonlinear problems with Dirichlet, mixed and Neumann boundary conditions defined on finite intervals. This paper introduces a modification of LT-HPM, the Non-linearities Distribution Laplace Transform-Homotopy Perturbation Method (NDLT-HPM), which will show better results for the case of linear and non-linear differential equations with non polynomial nonhomogeneous terms. The case of equations with boundary conditions on infinite intervals, has been studied in some articles, and corresponds often to problems defined on semi-infinite ranges (Aminikhah 2011; Khan et al. 2011). However the methods of solving these problems, are different from those presented in this paper (Filobello-Nino et al. 2013). As it is widely known, the importance of research on nonlinear differential equations is that many phenomena, practical or theoretical, are of nonlinear nature. In recent years, several methods focused to find approximate solutions to nonlinear differential equations, as an alternative to classical methods, have been reported, such those based on: variational approaches (Assas 2007; He 2007; Kazemnia et al. 2008; Noorzad et al. 2008), tanh method (Evans & Raslan 2005), exp-function (Xu 2007; Mahmoudi et al. 2008), Adomian’s Decomposition Method (ADM) (Adomian 1988; Babolian & Biazar 2002; Kooch & Abadyan 2012; Kooch & Abadyan 2011; Vanani et al. 2011; Chowdhury 2011; Elias et al. 2000), parameter expansion (Zhang & Xu 2007), HPM (Aminikhan & Hemmatnezhad 2012; Aminikhah 2012; Filobello-Nino et al. 2013; Aminikhah 2011; Khan et al. 2011; Marinca & Herisanu 2011; He 1998; He 1999; He 2006a; Vazquez-Leal et al. 2014; Belendez et al. 2009; He 2000; El-Shaed 2005; He 2006b; Vazquez-Leal et al. 2012a; Ganji et al. 2008; Fereidon et al. 2010; Sharma & Methi 2011; Biazar & Ghanbari 2012; Biazar & Eslami 2012; Araghi & Sotoodeh 2012; Araghi & Rezapour 2011; Bayat et al. 2014; Bayat et al. 2013; Vazquez-Leal et al. 2012b; Vazquez-Leal et al. 2012c; Filobello-Niño et al. 2012; Biazar & Aminikhan 2009; Biazar & Ghazvini 2009; Filobello-Nino et al. 2012; Khan & Qingbiao 2011; Madani et al. 2011; Ji Huan 2006; Feng et al. 2007; Mirmoradia et al. 2009; Vazquez-Leal et al. 2012; Vazquez-Leal et al 2013), Homotopy Analysis Method (HAM) (Rashidi et al. 2012a; Rashidi et al. 2012b; Patel et al. 2012; Hassana & El-Tawil 2011), and perturbation method (Filobello-Nino et al. 2013; Holmes 1995; Filobello-Niño et al. 2013b; Filobello-Nino et al. 2014) among many others. Also, a few exact solutions to nonlinear differential equations have been reported occasionally (Filobello-Niño et al. 2013a).

The case of Boundary Value Problems (BVPs) for nonlinear ODES includes, Michaelis Menten equation (Murray 2002; Filobello-Nino et al. 2014), that describes the kinetics of enzyme-catalyzed reactions, Gelfand’s differential equation (Filobello-Nino et al. 2013; Filobello-Niño et al. 2013b) which governing combustible gas dynamics, Troesch’s equation (Elias et al. 2000; Feng et al. 2007; Mirmoradia et al. 2009; Vazquez-Leal et al. 2012; Hassana & El-Tawil 2011; Erdogan & Ozis 2011), arising in the investigation of confinement of a plasma column by a radiation pressure, among many others.

In the same way, the theory of BVPs for linear ODES, is a well established branch of mathematics, with many applications. Between problems of interest, related to these equations, are found: The one-dimensional quantum problem, of a particle of mass m confined in a region of zero potential by an infinite potential at two points *x* = *a* and *x* = *b* (King et al. 2003), heat transfer equation (King et al. 2003), wave equation which describes for instance, transverse vibrations of a uniform stretched string between two fixed points, say *x* = *a* and *x* = *b* (Chow 1995; Zill Dennis 2012), the Laplace equation, which governs the temperature field corresponding to the steady state in a plate (Zill Dennis 2012), and so on. Generally, many problems expressed in terms of partial differential equations, give rise through method of separation of variables, to BVPs for linear ODES (Chow 1995; King et al. 2003; Zill Dennis 2012). From the above, it becomes a priority to investigate methods, to find handy analytical approximate solutions for linear and nonlinear ODES. With this end, we propose NDLT-HPM method, which as will be seen has good precision and requires a moderate computational work.

The paper is organized as follows. In Section 2, we introduce the standard HPM. Section 3, provides a basic idea of Nonlinearities Distribution Homotopy Perturbation Method (NDHPM). Section 4 introduces NDLT-HPM. Additionally Section 5 presents two cases study. Besides a discussion on the results is presented in Section 6. Finally, a brief conclusion is given in Section 7.

## Standard HPM

The standard HPM was proposed by Ji Huan He, it was introduced like a powerful tool to approach various kinds of nonlinear problems. The HPM is considered as a combination of the classical perturbation technique and the homotopy (whose origin is in the topology), but not restricted to small parameters as occur with traditional perturbation methods. For example, HPM requires neither small parameter nor linearization, but only few iterations to obtain highly accurate solutions (He 1998; He 1999).

To figure out how HPM works, consider a general nonlinear equation in the form
1

with the following boundary conditions
2

where *A* is a general differential operator, *B* is a boundary operator, *f*(*r*) a known analytical function and *Γ* is the domain boundary for *Ω. A* can be divided into two operators *L* and *N*, where *L* is linear and *N* nonlinear; so that (1) can be rewritten as
3

Generally, a homotopy can be constructed as (He 1998; He 1999)
4

or
5

where *p* is a homotopy parameter, whose values are within range of 0 and 1, *u*_0_ is the first approximation for the solution of (3) that satisfies the boundary conditions.

Assuming that solution for (4) or (5) can be written as a power series of *p*.
6

Substituting (6) into (5) and equating identical powers of *p* terms, there can be found values for the sequence *v*_0_, *v*_1_, *v*_2_,

When *p* → 1, it yields the approximate solution for (1) in the form
7

## Basic idea of NDHPM

(Vazquez-Leal et al. 2012b) introduced a modified version of HPM, which sometimes eases the solutions searching process for (3) and reduces the complexity of solving differential equations in terms of power series.

As first step, a homotopy of the form (Vazquez-Leal et al. 2012b) is introduced
8

or
9

It can be noticed that the homotopy function (8) is essentially the same as (4), except for the non-linear operator *N* and the non homogeneous function *f*, which contain embedded the homotopy parameter *p*. The standard procedure for the HPM is used in the rest of the method.

We propose that (He 1998; Vazquez-Leal et al. 2012b)
10

When *p* → 1, it is expected to get an approximate solution for (3) in the form
11

## Non-linearities distribution Laplace transform-homotopy perturbation method (NDLT-HPM)

A way to introduce, NDLT-HPM is assume that NDLT-HPM follows the same steps of NDHPM until (9), next we apply Laplace transform on both sides of homotopy equation (9), to obtain
12

(more generally, one could substitute *f*(*r*, *p*) in (8) by another function *g*(*r*, *p*) such that, *g*(*r*, *p*) → *f*(*r*) when *p* → 1, see cases study above).

Using the differential property of L.T, we have (Murray 1988)
13

or
14

applying inverse Laplace transform to both sides of (14), we obtain
15

Assuming that the solutions of (3) and *f*(*r*, *p*) can be expressed as a power series of *p*1617

Then substituting (16) and (17) into (15), we get
18

comparing coefficients of *p*, with the same power leads to
19

Assuming that the initial approximation has the form: *U*(0) = *u*_0_ = *α*_0_, *U*′(0) = *α*_1_,.., *U*^*n* − 1^(0) = *α*_*n* − 1_; therefore the exact solution may be obtained as follows
20

LT-HPM is derived in a similar way to NDLT-HPM, the difference is that in the first case the Laplace transform applies to (5) instead of (9). From here on, takes place in essence the same procedure followed by NDLT-HPM (12), (13), (14), (15), (16), (17), (18) and (19) (Aminikhan & Hemmatnezhad 2012; Aminikhah 2012; Filobello-Nino et al. 2013; Aminikhah 2011).

## Cases study

Next, NDLT-HPM, and LT-HPM are compared with the following two cases study

CASE STUDY 1

We will find an approximate solution the following nonlinear second order ordinary differential equation
21

Method 1 Employing LT-HPM

To obtain an approximate solution for (21) by applying the LTHPM method, we identify
2223

where prime denotes differentiation respect to *x*.

To solve approximately (21), first we expand the exponential term, resulting
24

We construct the following homotopy in accordance with (4)
25

or
26

where we have kept three terms of Taylor series.

Applying Laplace transform to (26) we get
27

As it is explained in (Murray 1988), it is possible to rewrite (27) as
28

where we have defined *Y*(*s*) = *ℑ*(*y*(*x*)).

After applying the initial condition *y*(0) = 0, the last expression can be simplified as follows
29

where, we have defined *A* = *y*′(0).

Solving for *Y*(*s*) and applying Laplace inverse transform *ℑ*^− 1^30

Next, suppose that the solution for (30) has the form
31

and choosing
32

as the first approximation for the solution of (21) that satisfies the condition *y*(0) = 0.

Substituting (31) and (32) into (30), we get
33

Equating terms with identical powers of *p*, we obtain
3435363738

From above we solve for *ν*_0_(*x*), *ν*_1_(*x*), *ν*_2_(*x*),.. we obtain
3940414243

and so on.

By substituting solutions (39), (40), (41), (42) and (43) into (20) results in a fourth order approximation
44

In order to calculate the value of *A*, we require that (44) satisfies the boundary condition *y*(1) = 2, so that we obtain
45

Method 2 Employing NDLT-HPM

In accordance with NDLT-HPM, we propose the following homotopy
46

we see that (46) is not exactly of the form (8), but note that *g*(*x*, *p*) = *pe*^*px*^ → *e*^*x*^, if *p* → 1.

After expanding the exponential term, we obtain
47

or
48

Applying Laplace transform to (48), we get
49

it is possible to rewrite (49) as
50

where we have defined *Y*(*s*) = *ℑ*(*y*(*x*)).

Applying the initial condition *y*(0) = 0, (50) can be simplified as follows
51

where, we have defined *A* = *y*′(0).

Solving for *Y*(*s*) and applying Laplace inverse transform *ℑ*^− 1^52

Assuming that the solution for (52) has the form
53

and choosing
54

as the first approximation for the solution of (21) that satisfies the condition *y*(0) = 0.

Substituting (53) and (54) into (52), we get
55

Equating terms with identical powers of p, we obtain
5657585960

Solving the above equations for *ν*_0_(*x*), *ν*_1_(*x*), *ν*_2_(*x*) …, we obtain
6162636465

and so on.

By substituting solutions (61), (62), (63), (64) and (65) into (20) results in a fourth order approximation
66

In order to calculate the value of *A*, we require that (66) satisfies the boundary condition *y*(1) = 2, so that we obtain
67

Case study 2

We will find an approximate solution for the following linear third order ordinary differential equation with variable coefficients.
68

Method 1 Employing LT-HPM

To obtain a solution for (68) by applying the LT-HPM method, we identify
6970

where prime denotes differentiation respect to *x*.

To solve approximately (68), first we expand the trigonometric term, resulting
71

We construct the following homotopy in accordance with (4)
72

where we have kept just two terms of Taylor series,

or
73

Applying Laplace transform to (73), we get
74

In accordance with (Murray 1988), it is possible to rewrite (74) as
75

Applying the initial conditions *y*(0) = 0 and *y*′(0) = 1, (75) adopts the following form
76

where, we have defined *A* = *y*″(0).

Solving for *Y*(*s*) and applying Laplace inverse transform *ℑ*^− 1^77

Assuming that the solution for (77) has the form
78

and choosing
79

let be the first approximation for the solution of (68) that satisfies the initial conditions *y*(0) = 0 and *y*′(0) = 1.

Substituting (78) and (79) into (77), we get
80

Equating terms with identical powers of *p*, we obtain
8182838485

From above we solve for *ν*_0_(*x*), *ν*_1_(*x*), *ν*_2_(*x*) …, we obtain
8687888990

and so on.

By substituting solutions (86), (87), (88), (89) and (90) into (20) results in a fourth order approximation
91

In order to calculate the value of *A*, we require that (91) satisfies the boundary condition *y*(1) = 2, so that we obtain
92

Method 2 Employing NDLT-HPM

In accordance with NDLT-HPM, it is possible to propose the following homotopy (see (9))
93

where we have defined
94

with the property
95

after expanding the two first terms of sin function, we obtain
96

or
97

Applying Laplace transform to (97) we get
98

it is possible to rewrite (98) as
99

where once again, we have defined *Y*(*s*) = *ℑ*(*y*(*x*)).

Applying the initial conditions *y*(0) = 0, and *y*′(0) = 1, (99) can be simplified as follows
100

where, we have defined *A* = *y*″(0).

Solving for *Y*(*s*) and applying Laplace inverse transform *ℑ*^− 1^101

Next, we assume a series solution for *y*(*x*), in the form
102

let
103

be the first approximation for the solution of (68) that satisfies the initial conditions *y*(0) = 0 and *y*′(0) = 1.

Substituting (102) and (103) into (101), we get
104

On comparing the coefficients of like powers of *p* we have
105106107108109

Performing the above operations for *ν*_0_(*x*), *ν*_1_(*x*), *ν*_2_(*x*) …, we obtain
110111112113114

and so on.

By substituting solutions (110), (111), (112), (113) and (114) into (20) and calculating the limit when *p* → 1, results in a fourth order approximation
115

In order to calculate the value of *A*, we require that (115) satisfies the boundary condition *y*(1) = 2, resulting an equation for *A*, from which we obtain the following result
116

## Discussion

This work showed the accuracy of NDLT-HPM in solving ordinary differential equations with nonhomogeneous non-polynomial terms and finite boundary conditions, and it can be considered as a continuation of (Filobello-Nino et al. 2013) where in principle, LT-HPM already provided the possibility of solving problems, with the nonhomogeneities mentioned in this study (Aminikhan & Hemmatnezhad 2012; Aminikhah 2012; Filobello-Nino et al. 2013; Aminikhah 2011), but were not carried out. One way to introduce LT-HPM to this kind of problems, is directly apply the Laplace transform to the homotopy equation (5) and then following a procedure identical to that applied in (Filobello-Nino et al. 2013) (see also (12), (13), (14), (15), (16), (17), (18) and (19)), although a possible difficulty is that, the mathematical procedure becomes long and cumbersome, depending on the function (see (4)). It may even happen that, the method does not work if the Laplace transform does not exist. Another possibility, which was followed in this study is to use a few terms of the Taylor series of *f*. Although the Taylor expansion allowed apply the LT-HPM method, we noted that a possible drawback of this strategy is that it may not produce handy approximate solutions, containing more computational requirements. For comparison purposes, we will consider for both cases study, that the “exact” solution is computed using a scheme based on a trapezoid technique combined with a Richardson extrapolation as a build-in routine from Maple 17. Moreover, the mentioned routine was configured using an absolute error (A.E.) tolerance of 10^− 12^ .

In this study was considered the exponential and sine functions respectively and we saw that the process of getting approximate solutions by using LT-HPM, was unnecessarily long and complicated. In order to deal with the above mentioned problems, this paper introduced NDLT-HPM.

**Figure 1:**
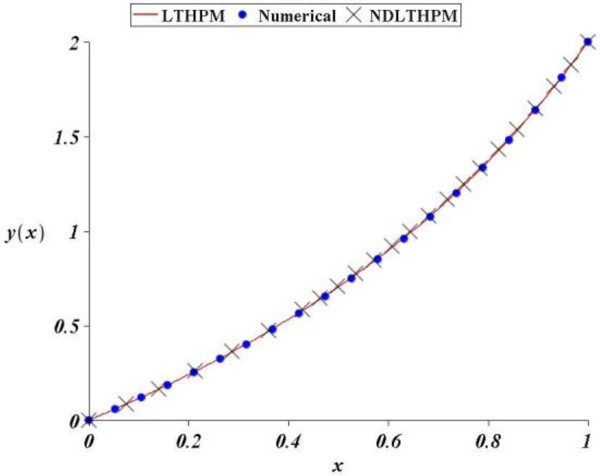
**Comparison between numerical solution of (**21**) and LT-HPM, NDLT-HPM approximations (**44**), (**66**).**

**Figure 2:**
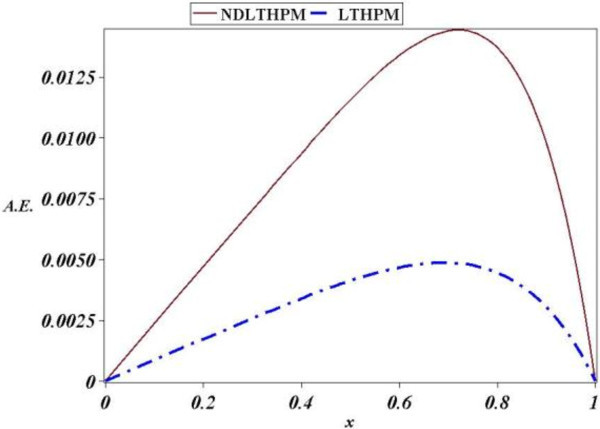
**Absolute Error (A.E.) between numerical solution of (21) and LT-HPM, NDLT-HPM approximations (**44**), (**66**).**

At the first place, we studied a nonlinear second order ordinary differential equation with an exponential nonhomogeneous non-polynomial term. This example, proposed the application of LT-HPM, keeping only three terms of the Taylor expansion of *e*^*x*^ from where it was obtained the fourth order approximation (44) and although the final approximation had good accuracy (Figure 1), it is clear that the procedure of solution was cumbersome.On the other hand, the application of NDLT-HPM to the same problem is outlined in (61), (62), (63), (64) and (65) and can be seen by inspection that exist a considerable saving of computational effort, even NDLT-HPM approximation (66) not only turned out to be clearly shorter than (44), but from the Figures 1 and 2 is scarcely less accurate.

**Figure 3:**
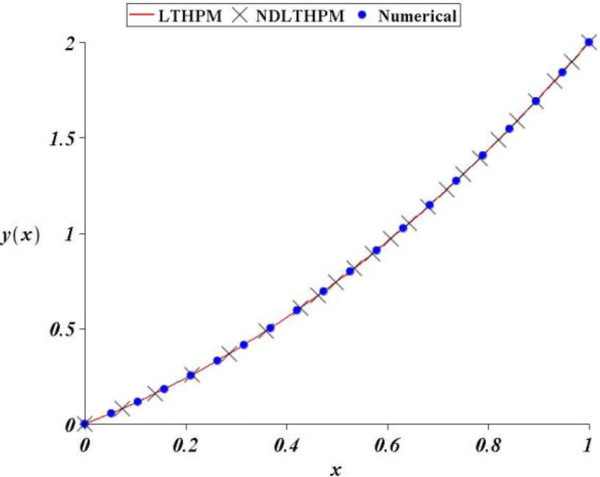
**Comparison between numerical solution of (**68**) and LT-HPM, NDLT-HPM approximations (**91**), (**115**).**

**Figure 4:**
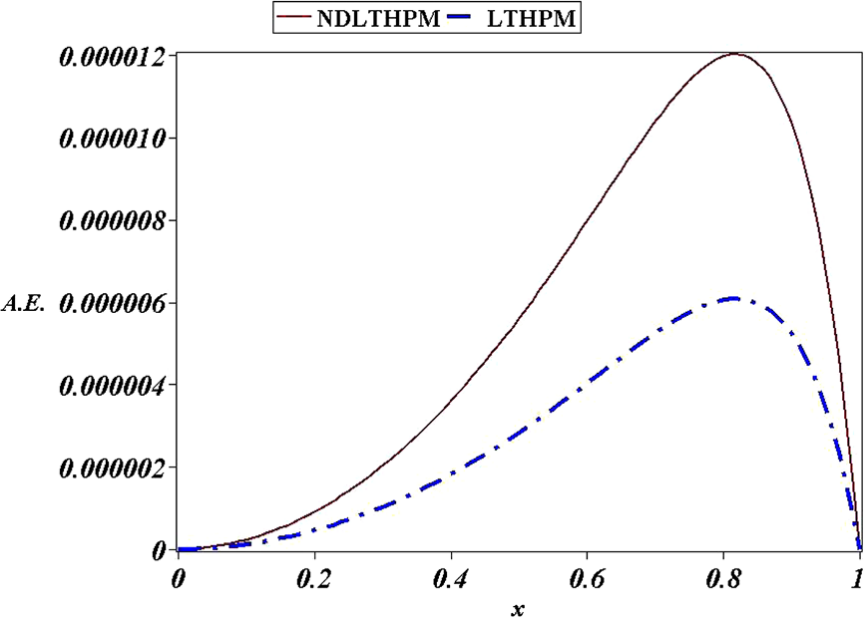
**Absolute Error (A.E.) between numerical solution of (**68**) and LT-HPM, NDLT-HPM approximations (**91**), (**115**).**

Next, we found an approximate solution for the linear third-order equation of variable coefficients, (68) and although we kept only two terms of the Taylor series of sin(*x*), LT-HPM got a precise approximation (see Figure 3). Iterations (86), (87), (88), (89) and (90) for LT-HPM show again a long computational process compared to NDLT-HPM (110), (111), (112), (113) and (114) but the Figure 3 reveals that in fact, both methods are highly accurate, and although NDLT-HPM is handier its absolute error is again slightly less accurate.In more precise terms, Figure 2 shows that LT-HPM, NDLT-HPM approximations (44) and (66), are accurate analytical approximate solutions for (21). The biggest absolute error (A.E) of LT-HPM and NDLT-HPM turned out to be 0.000006 and 0.000012 respectively, while from Figure 4 we conclude that the second case study got for the same methods, the values of A.E 0.004 and 0.014. In Spite of this it is noted that NDLT-HPM got a slightly small loss of accuracy with respect to LT-HPM, the comparison of computational effort for both methods leads to the conclusion that NDLT-HPM is more compact, handy and easy to compute, therefore it is an useful tool with good accuracy in the search of solutions for ODES of the type already mentioned.

Finally, we observe that the proposed homotopy formulations (46) and (93) are something different from the original propose in (9) (Vazquez-Leal et al. 2012b), which shows the richness and flexibility of NDHPM and of course of NDLT-HPM. Indeed, the mentioned homotopies were formulated in this way, with the aim that the computational work was reduced considerably, without losing a great precision in the results. Although it is possible to consider other variants of the homotopy given in (9), the key point is that, in the limit when *p* → 1, the homotopy equation is reduced to the differential equation to be solved.

## Conclusions

In this paper NDLT-HPM was introduced as a useful strategy capable of supporting approximate methods, simplifying mathematical iterative procedure, building handy and easy computable expressions in comparison with LT-HPM, in the search for analytical approximate solutions for linear and nonlinear ordinary differential equations with finite boundary conditions, for the case of equations with nonhomogeneous non-polynomial terms. Moreover, the accuracy of the proposed approximate solutions are in good agreement with the exact solutions.

Such as it was explained, NDLT-HPM method expresses the problem of finding an approximate solution for an ordinary differential equation, in terms of solving an algebraic equation for some unknown initial condition (Filobello-Nino et al. 2013). Figure 1 through Figure 4 show how good this procedure is in the search for analytical approximate solutions with good precision, and a moderate computational effort. In addition, just as with LT-HPM, the proposed method does not need to solve several recurrence differential equations. From all the above, we conclude that NDLT-HPM method is a reliable and precise tool in practical applications.
